# Synthetic Megavoltage Cone Beam Computed Tomography Image Generation for Improved Contouring Accuracy of Cardiac Pacemakers

**DOI:** 10.3390/jimaging9110245

**Published:** 2023-11-08

**Authors:** Hana Baroudi, Xinru Chen, Wenhua Cao, Mohammad D. El Basha, Skylar Gay, Mary Peters Gronberg, Soleil Hernandez, Kai Huang, Zaphanlene Kaffey, Adam D. Melancon, Raymond P. Mumme, Carlos Sjogreen, January Y. Tsai, Cenji Yu, Laurence E. Court, Ramiro Pino, Yao Zhao

**Affiliations:** 1 MD Anderson Cancer Center UTHealth Houston Graduate School of Biomedical Sciences, The University of Texas, Houston, TX 77030, USA; 2 Department of Radiation Physics, The University of Texas MD Anderson Cancer Center, Houston, TX 77030, USA; 3 Department of Anesthesiology and Perioperative Medicine, Division of Anesthesiology, Critical Care Medicine and Pain Medicine, The University of Texas MD Anderson Cancer Center, Houston, TX 77030, USA; 4 Department of Radiation Oncology, Houston Methodist Hospital, Houston, TX 77030, USA

**Keywords:** CT/CBCT artifacts, machine learning, generative adversarial networks, pacemaker visualization

## Abstract

In this study, we aimed to enhance the contouring accuracy of cardiac pacemakers by improving their visualization using deep learning models to predict MV CBCT images based on kV CT or CBCT images. Ten pacemakers and four thorax phantoms were included, creating a total of 35 combinations. Each combination was imaged on a Varian Halcyon (kV/MV CBCT images) and Siemens SOMATOM CT scanner (kV CT images). Two generative adversarial network (GAN)-based models, cycleGAN and conditional GAN (cGAN), were trained to generate synthetic MV (sMV) CBCT images from kV CT/CBCT images using twenty-eight datasets (80%). The pacemakers in the sMV CBCT images and original MV CBCT images were manually delineated and reviewed by three users. The Dice similarity coefficient (DSC), 95% Hausdorff distance (HD95), and mean surface distance (MSD) were used to compare contour accuracy. Visual inspection showed the improved visualization of pacemakers on sMV CBCT images compared to original kV CT/CBCT images. Moreover, cGAN demonstrated superior performance in enhancing pacemaker visualization compared to cycleGAN. The mean DSC, HD95, and MSD for contours on sMV CBCT images generated from kV CT/CBCT images were 0.91 ± 0.02/0.92 ± 0.01, 1.38 ± 0.31 mm/1.18 ± 0.20 mm, and 0.42 ± 0.07 mm/0.36 ± 0.06 mm using the cGAN model. Deep learning-based methods, specifically cycleGAN and cGAN, can effectively enhance the visualization of pacemakers in thorax kV CT/CBCT images, therefore improving the contouring precision of these devices.

## 1. Introduction

Cancer and heart disease stand as the two leading causes of death in the United States [1]. As the incidence of both diseases continues to rise, more radiation oncology patients will present with cardiac-implantable electronic devices (CIEDs), posing challenges for radiation therapy workflows. One challenge is the image quality of the computed tomography (CT) and cone beam CT (CBCT) images used for radiation planning and daily adaptive planning. The presence of metallic devices, characterized by a high atomic number and density, significantly attenuates X-ray beams, resulting in beam-hardening. This phenomenon entails the preferential absorption of lower-energy X-rays, leading to an overestimation of tissue density and the formation of streaks or shadows around the device. Moreover, metal objects can scatter X-rays, contributing to streak artifacts and distorting the representation of surrounding tissues. The artifacts introduced by Cardiovascular Implantable Electronic Devices (CIEDs) have the potential to impede anatomical clarity, increasing variability in device contouring among different experts. Contouring these devices and overriding their density is a crucial step in the radiotherapy treatment planning process, ensuring accurate dose calculations, precise treatment delivery, and overall patient safety. This impact is further influenced by the diverse shapes and sizes of CIEDs used in medical procedures, leading to varying degrees of severity. Extensive research across multiple disease sites in radiation oncology has thoroughly explored the negative effects of CT artifacts on contouring and dose calculation [2,3,4]. In a study by DiFilippo et al., implantable cardioverter defibrillators caused streaking artifacts that spanned across 30–50 mm of tissue equivalent [5]. In addition, the presence of metal artifacts in CT scans hinders the diagnostic accuracy and overall image quality, affecting the visualization of various tissues and their surroundings [6]. Addressing and resolving these artifacts are pivotal steps for ensuring the reliability and efficacy of CT imaging, affecting not only diagnostic and treatment planning but overall patient care.

Current solutions for reducing artifacts in CT imaging utilize a combination of advanced techniques. One such technology is dual-energy CT, which is characterized by data capture at two distinct energy spectra. Virtual monochromatic pictures created at high-kiloelectron volt levels are known to lessen beam-hardening effects. In addition, approaches to the commercial reduction in metal artifacts have been developed and tested for their efficacy. One study compared the quantitative measures of four manufacturers’ metal artifact reduction (MAR) approaches to CT imaging with a self-made acrylic phantom; the effect was substantial with three types of metal implants but not with dental fillings because of their high density. These results demonstrate the importance of selecting the right metal artifact reduction approach based on the type and size of the metal implant for optimal artifact reduction in CT imaging [7]. Some of the latest advancements in this domain involve deep learning-based tools. Lu et al. employed a novel reinforced transformer network model to enhance vessel imaging in coronary CT angiography, showcasing the adaptability of deep learning architectures to address specific challenges [8]. Other work involves the use of convolutional neural networks to eliminate motion-induced artifacts and dental artifacts on CT scans [9,10]. While state-of-the-art commercial MAR methods are known for their accuracy, recent research by Huang et al. has revealed that deep learning MAR methods like generative adversarial networks (GANs) surpass these tools in performance [11].

In recent years, GANs have been investigated to address the problem of medical image artifacts in dose calculation for treatment planning. Research has been conducted into the use of GANs in the accurate generation of synthetic CTs [12,13]. GANs have also been investigated for multi-modal medical image generation (e.g., MR to CT, CT to PET, or CBCT to CT), dose reduction, and image artifact removal [14,15,16,17,18]. Gomi et al. reported adequate artifact removal in digital tomosynthesis at up to 55% radiation dose reduction after the extensive model optimization of GAN architectures to minimize the mean squared error and structural similarity [19]. Another study by Liao et al. reduced metal artifacts on CT and CBCTs by correcting the affected regions through joint projection–sinogram correction and adversarial learning [20]. While GANs have been employed in various MAR research contexts, their application to addressing artifacts caused by cardiac pacemakers in CT/CBCT images, which obscure the outlines of these devices, has not been explored.

In this study, we propose a novel method for improving the visualization of pacemakers on kV CT/CBCT images using megavoltage (MV) CBCT to synthetically produce scans of similar quality. While CT provides the golden standard of image quality, cone-beam CT (CBCT) is more effective for patient positioning and verifying radiation dose because it is faster. It can also be incorporated in a linear accelerator that delivers radiation treatment which reduces misalignment issues when moving from imaging to treatment units. MV CBCT is more accurate for imaging high Z materials than kilovoltage (kV) CBCT is; the higher energy leads to less scatter from the devices and therefore the absence of streaking artifacts on the scan. To the best of our knowledge, this is the first feasibility investigation of the use of GAN-based models to produce synthetic MV (sMV) CBCT images from kV CT/CBCT images to help visualize pacemakers on CT images and hence better contour them. The primary contributions of this paper include (a) investigating and demonstrating the efficacy of GAN-based deep learning models in enhancing the visualization of pacemakers on kV CT/CBCT images; (b) introducing MV CBCT for model development, enabling improved pacemaker visualization through the generation of synthetic MV CBCT images.

## 2. Materials and Methods

In this study, we created, trained, and compared two deep learning models. The goal was to improve the visualization of pacemakers on kV CT/CBCT images with streaking artifacts caused by this type of device. For this purpose, we collected and matched kV CT, MV CBCT, and kV CBCT images. The following sections detail the specifics on data acquisition, pre-processing, and model training and evaluation.

### 2.1. Data Acquisition

CT images were acquired using a Siemens SOMATOM CT scanner (USA) with a 2 mm slice thickness, 120 kVp, and 52.5 mAs. Chest CT images were reconstructed using SAPHIRE strength 3, kernel Qr40, and artifacts were suppressed with iterative metal artifact reduction (iMAR) on the pacemaker setting. A Varian Halcyon was used to acquire MV CBCT and kV CBCT images. The MV CBCT images were acquired under high-quality mode (10 MU delivered) via continuous gantry rotation from 260° to 100°. The kV CBCT images were acquired under thorax mode enhanced with iterative reconstruction.

**Figure 1:**
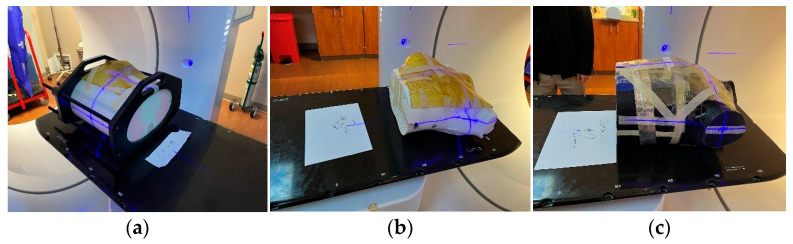
Setups of each phantom: (**a**) CIRS, (**b**) 3D-printed phantom, and (**c**) Rando at the Halcyon table.

Ten pacemakers from seven vendors, EliTE II (Medtronic, Minneapolis, MN, USA), Discovery II (Guidant Corporation, Indianapolis, IN, USA), InSync Sentry (Medtronic, USA), INSIGINIA I Plus (Guidant Corporation, USA), VENT AK (Medtronic, USA), ACTIVITRAX(Medtronic, USA), and St. Jude Medical (Saint Paul, MN, USA), and four thorax phantoms, Rando, CIRS [21,22], and two 3D-printed chest wall phantoms [23], were included in our study. Thirty-five pacemaker/phantom combinations were imaged with MV CBCT, kV CBCT, and CT, resulting in 3 images, with a total of 105 images. Each of the pacemakers were placed flat on the chest area of the phantoms, securely covered by a 5 mm bolus (Figure 1 shows the setups for the different phantoms). A phantom and a pacemaker were assembled before obtaining the CBCT scans and then transferred to the CT room to acquire CT images. Maintaining consistent and accurate setup positions between the CBCT and CT scans was of paramount importance. To achieve this, a systematic approach was employed. Firstly, during the initial setup, both sides of the phantoms were meticulously marked to serve as reference points. This marking procedure ensured a reliable and reproducible setup. Subsequently, to minimize disturbances and maintain alignment integrity, the phantoms, each with their associated pacemaker, were transported to the CT room using specialized carts. Upon reaching the CT room, the phantoms were arranged in accordance with the previously established markings, ensuring a seamless transition from CBCT to CT imaging.

### 2.2. Data Preprocessing

Although the phantoms were marked and placed carefully to ensure minimal differences in setup between CBCT and CT acquisitions, misalignments between the MV CBCT and CT images were unavoidable. Therefore, rigid registration between MV CBCT and CT images was needed to obtain paired data for the conditional GAN model training. On the other hand, the kV CBCT and MV CBCT images were acquired at the same position without moving the phantoms, and the kV CBCT images had similar image intensities as the CT images. Therefore, registration between the kV CBCT and MV CBCT was not needed.

To improve the accuracy of the registration, we registered CT to kV CBCT images. We utilized SimpleITK [17] to implement a rigid registration method that minimizes the Mattes Mutual Information between kV CBCT and CT scans through the application of a gradient optimization algorithm. A multi-resolution framework with a shrink factor of 4, 2, and 1 was employed to speed up the registration process. The aligned CT, kV CBCT, and MV CBCT images were then cropped to the overlapped region. Subsequently, the CT and kV CBCT images were clipped with CT numbers of −1000 to 2000 Hounsfield Units (HU), and the MV CBCT image was clipped with −1000 to 400 HU.

To generate image patches for training the deep learning models, body masks were generated on MV CBCT images using a thresholding method. To limit the air region and maximize the amount of the patient’s body in each patch, the patch centers were restricted to within the body, using the body mask as a guide. The 2D 256 × 256 patches were extracted for model training.

### 2.3. Model Training

**Figure 2:**
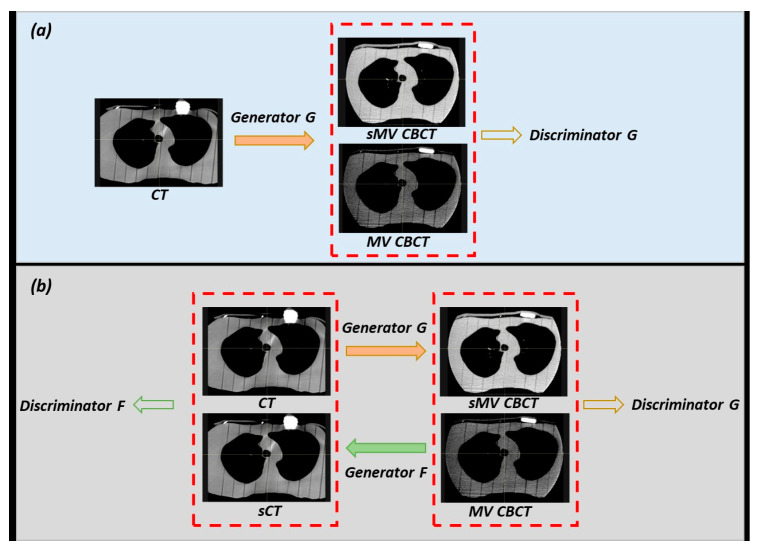
Illustration of (**a**) the conditional GAN model: one generator and one discriminator and (**b**) the cycleGAN model: two generators and two discriminators. sMV CBCT: synthetic MV CBCT; sCT: synthetic CT.

In this study, two deep learning models, conditional GAN (cGAN) [24] and cycleGAN [25,26], were used to translate the CT/kV CBCT images to MV CBCT. Their frameworks are shown in Figure 2. The generator and discriminator networks in GANs work in opposition to each other, with the generator aiming to generate realistic synthetic images and the discriminator trying to distinguish them from real images. The training process involves improving the ability of generators to create realistic synthetic images while simultaneously improving the ability of discriminators to accurately differentiate between synthetic and real images.

As shown in Figure 2, the cGAN model consists of one generator (generator G) and one discriminator (discriminator G), while the cycleGAN model consists of two generators (generator G and F) and two discriminators (discriminator G and F). In the training stage, the generators and discriminators were trained simultaneously to achieve an optimal solution in an adversarial manner. The generators used in this study are Residual Neural Network (ResNet) [27] consisting of three convolutional layers, nine residual blocks, and three transposed convolutional layers. The purpose of this network is to generate synthetic MV CBCT images of the same dimensions as the input (CT/kV CBCT). The layers are sequentially followed by instance normalization and Leaky Rectified Linear Unit (ReLU) activation [28]. In addition, the discriminator networks consist of five convolutional layers, each of which is followed by Leaky ReLU activation to estimate the authenticity of images at a sub-regional level. After the models have been trained, the generator can directly generate sMV CBCT images by inputting CT/kV CBCT images.

### 2.4. Model Evaluation

To evaluate the performance of the cGAN and cycleGAN models, 28 datasets (80%) were randomly selected for training and validation, and the remaining 7 (20%) were used for model testing. In total, four models were trained to generate the sMV CBCT images, including (1) cycleGAN (CT-to-MV CBCT), (2) cGAN (CT-to-MV CBCT), (3) cycleGAN (kV CBCT-to-MV CBCT), and (4) cGAN (kV CBCT-to-MV CBCT). In this study, paired data were utilized to train cycleGAN and cGAN to ensure a fair comparison, even though cycleGAN does not necessitate such paired data.

For each test patient, two sMV CBCT images were generated from their corresponding CT or kV CBCT image using the cGAN and cycleGAN models. To evaluate and compare the performance of the two models, a comprehensive analysis was carried out involving both quantitative and qualitative assessments of the sMV CBCT images generated by each model. To be more specific, the pacemakers in the sMV CBCT images and original MV CBCT image were manually delineated and reviewed by three users. The Dice similarity coefficient (DSC), surface DSC (1 mm), 95 percentile Hausdorff distance (HD95), and mean surface distance (MSD) were calculated to quantify the similarities between contours derived from the sMV CBCT images and original MV CBCT image.

The Dice similarity coefficient measures the region of overlap relative to the union of the contour on the original image and the one delineated on the synthetic image, with a perfect match indicated by a value of 1. In contrast, the Surface Dice Coefficient compares surfaces, considering points within 1 mm as in agreement. The Hausdorff distance captures the maximum distance from one point in one contour to its closest point in the other contour, and the 95th percentile accounts for outliers, offering a representative measure of the bulk of the data. The mean surface distance is the average of all distances from points on the ground truth contour to the evaluated one. These geometric metrics collectively provide a comprehensive evaluation, with high DSC and SDSC of 1 mm (close to 1) and low HD95 and MSD (close to 0) indicating favorable results.

To statistically compare the performance of the cGAN versus the CycleGAN models and determine the superior model’s performance, a two-tailed Wilcoxon rank-sum test was performed. A *p*-value lower than 0.05 suggests that there is enough evidence to conclude that there is a statistically significant difference between the two models. This analysis aimed to provide a rigorous assessment and insight into the effectiveness of the models in generating sMV CBCT images.

## 3. Results

### 3.1. Visual Inspection

**Figure 3:**
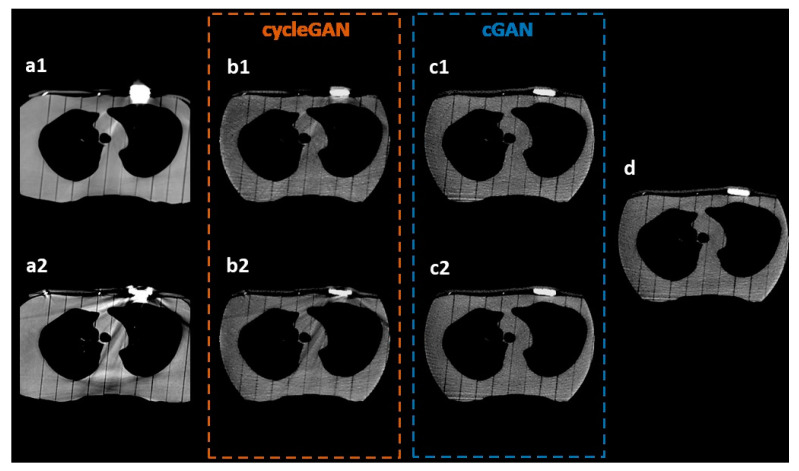
Visual inspection of the artifact correction in synthetic images. The synthetic images generated by different models were compared with the ground-truth real MV CBCT images. Axial views of (**a1**, **a2**) original CT or kV CBCT images, (**b1**, **b2**) synthetic images generated by cGAN models, (**c1**, **c2**) synthetic images generated by cycleGAN models, and (**d**) ground-truth MV CBCT image.

The efficacy of the trained models in enhancing the visualization of pacemakers was evaluated by comparing the sMV images generated by cGAN and cycleGAN with the ground-truth MV CBCT images. The results of the visual comparison are presented in Figure 3, including the axial views of the original CT/kV CBCT images (a1, a2), the corresponding real MV CBCT image (d), and the synthetic images generated by cycleGAN (b1, b2) and cGAN (c1, c2).

The visual inspection demonstrated a considerable improvement in the visualization of pacemakers on the sMV CBCT images generated by both the cycleGAN and cGAN models compared to the original kV CT/CBCT images. As shown in Figure 3, it was challenging to discern the actual shape of pacemakers in the original CT (a1) and kV CBCT (a2) images.

The synthetic images generated by cycleGAN (b1, b2) and cGAN (c1, c2) depicted the pacemakers more distinctively, making their shape and presence more easily recognizable. Although minor artifacts remained in the synthetic images, especially on the cycleGAN-generated images, the overall visualization of the pacemakers was substantially enhanced. The results suggest that the use of cycleGAN and cGAN models can effectively improve the visualization of pacemakers, allowing easier identification and segmentation in radiation therapy.

### 3.2. Model Comparison

**Figure 4:**
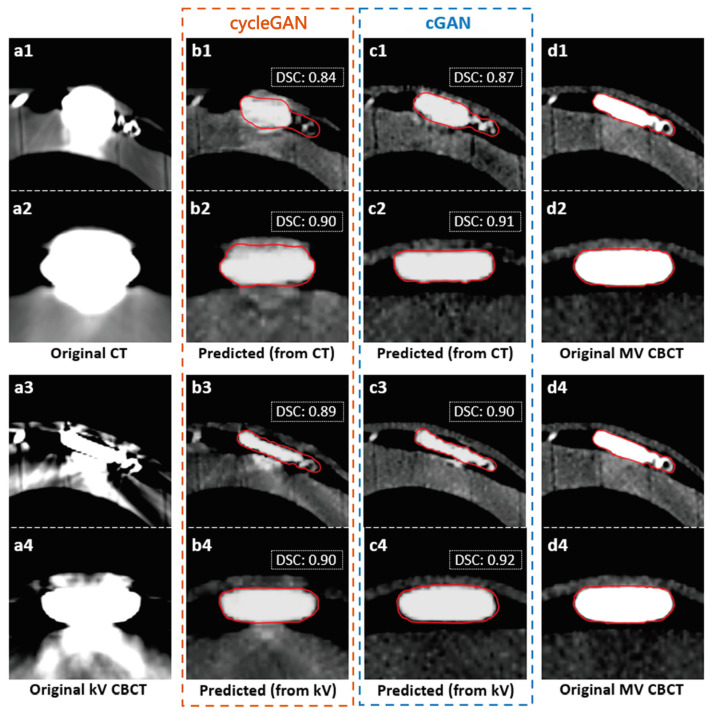
Comparison between the contours on synthetic and real MV CBCT imagesRed lines indicate the pacemaker contours delineated and reviewed by three users. The Dice similarity coefficient (DSC) results comparing the contours between the original and synthetic images are included for reference.

As depicted in Figure 3, the results of the visual comparisons indicate that the cGAN model outperforms the cycleGAN model in improving the visualization of pacemakers. Specifically, the cGAN models generate sMV CBCT images (c1, c2) that more closely resemble the actual shapes of pacemakers, as seen in the original MV CBCT image (d). To effectively compare the performance of cGAN and cycleGAN, the pacemakers were manually delineated on both sMV and real CBCT images. The comparison of these contours is shown by the red lines in Figure 4. The results show that, for the same input kV CT/CBCT images, cGAN models consistently generated more accurate pacemaker shapes in sMV CBCT images, as illustrated by the comparison of their contours.

To further evaluate the performance of the two models, a quantitative analysis was conducted that involved computing the DSC, surface DSC (1 mm), HD95, and MSD between the manual contours of the pacemakers in the sMV CBCT images and the original MV CBCT images. The DSC values of corresponding cases are presented in Figure 4 for reference. Table 1 provides a summary of the average results over all test cases. cGAN models achieved DSCs of 0.91 and 0.92 for CT-to-MV and kV-to-MV, while cycleGAN only achieved 0.89 and 0.91, respectively. The results revealed that cGAN outperformed cycleGAN in all evaluation metrics for both CT-to-MV and kV CBCT-to-MV scenarios, which was consistent with the results of the visual comparison. However, the two-tailed Wilcoxon rank-sum test showed no significant improvement in the quantitative results of the cGAN model (*p* > 0.05).

## 4. Discussion

In this study, GAN-based models were shown to be effective tools for improving pacemaker visualization in kV imaging, facilitating simplified contouring of the device. Furthermore, through quantitative analysis and visual inspection, cGAN models demonstrated superior performance compared to cycleGAN models. This was mainly attributed to the use of paired data during the development of the models. The cGAN models benefitted from the paired data as the models were able to learn direct mapping from kV CBCT/CT to MV CBCT images, which led to a more effective correction of pacemaker visualization in the final sMV CBCT images. However, acquiring such data can be challenging in clinical practices. In such cases, the cycleGAN model can be trained to provide adequate results in the enhancement of the pacemaker visualization in thorax kV CT/CBCT images.

**Figure 5:**
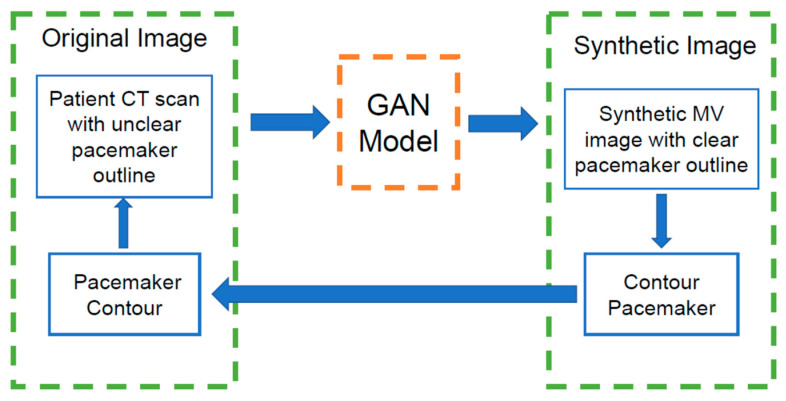
Workflow of the potential benefit of using GAN-based models to produce synthetic MV (sMV) CBCT images from planning CT images in a clinical setting.

One advantage of our findings is that the models used in this study may be translatable to actual patients’ scans. By using GAN-based models to produce synthetic MV CBCT from kV CTs in a clinical setting, experts might be able to easily contour the pacemaker and its surroundings with fewer streaks obstructing the outline of the device; Figure 5 illustrates this process.

Consequently, this approach can reduce the time required for implant delineation and enhance the accuracy of the process, thereby improving patient outcomes. Another advantage of this method is its potential benefit in adaptive treatment planning; the enhanced visualization of the implant on daily CBCT scans might make it easier and faster to re-plan when needed. This work can also be easily translatable to different implant devices, such as defibrillators, cardiac loopers, and breast expanders.

In a study similar to ours, Gao et al. aimed to enhance CBCT image quality by generating synthetic CT images, enabling their use in dose calculations and adaptive treatment [29]. They conducted a comparative analysis of deep learning models, including U-Net, cycleGAN, cycAT, and cascadeGAN. Their findings indicated that while cycleGAN effectively reduced artifacts, some streaking artifacts persisted. In contrast, our work focuses directly on mitigating pacemaker-induced artifacts, eliminating the need for artificial artifact creation, thus achieving greater realism. Moreover, our approach addresses artifact correction not only in CT but also in CBCT images. Another study by Cao et al. explored the use of cycleGAN for synthetic CT generation to reduce denture artifacts [30]. However, their approach used artifact-free CT data paired with MVCBCT, which may not adequately replicate pacemaker-induced artifacts in the thorax region due to significant anatomical differences compared to the head and neck region. Pennig et al. conducted research on reducing artifacts caused by cardiac implantable devices in CTs [31]. They experimented with various reconstruction kernels and determined that a combination of virtual monoenergetic images and metal artifact reduction kernels yielded the best quantitative and subjective artifact reduction results. However, the requirement for dual CT imaging limits the widespread applicability of virtual monoenergetic images in clinical settings.

It is important to note that our work primarily focuses on synthesizing MV CBCTs. This makes direct dosimetric comparisons unfeasible due to the inherent inaccuracies in Hounsfield Unit (HU) values in synthetic CBCTs. Our evaluation methods are tailored to improve the visualization of pacemakers in both CT and kV CBCT images, enhancing their suitability for contouring during treatment planning and daily adaptive planning, without requiring scatter quantification or complex dosimetric assessments.

Considering the rapid development in this field, more advanced GAN models are consistently becoming available. While we did not directly compare our models with the most recent GAN models in this study, we acknowledge that ongoing developments will lead to further improvements in this area. However, considering the positive outcomes of our work in enhancing pacemaker visualization, the enhancements achievable with more advanced models might be relatively modest.

One limitation of this study is the lack of real patient data in the training model. Further investigation will be required to assess the model’s accuracy on actual patients’ CTs, as the use of phantoms reduces anatomy variability. Another limitation is the use of a training and testing set from the same imaging devices with consistent imaging protocols and phantom anatomy. More testing is needed to evaluate the model on an external testing set.

In summary, our study has convincingly showcased the practical utility of GAN-based models in enhancing the visualization of pacemakers within kV CT and CBCT phantom images. The positive outcomes observed in our investigation strongly suggest the feasibility of applying this methodology in a clinical setting, particularly to ameliorate the contouring process. By successfully leveraging GAN-based models, we have demonstrated their effectiveness in enhancing the clarity and visibility of pacemakers within these imaging modalities. This not only underscores the potential for significant improvement in the precision of pacemaker delineation but also signifies the broader applicability of our findings to real-world clinical scenarios. The enhanced visualization achieved through GAN-based approaches holds promise for optimizing the accuracy and efficiency of contouring procedures, thereby contributing to improved clinical workflows and, ultimately, patient care.

## Figures and Tables

**Table 1:** Quantitative comparison of cGAN and cycleGAN for artifact correction. The averaged results over all test patients are summarized for each model. CT-to-MV model indicates the model that was trained on kV CT and outputs MV CBCT, and kV-to-MV indicates kV CBCT to MV CBCT. DSC: Dice similarity coefficient; HD95: 95th percentile Hausdorff distance; MSD: mean surface distance; cGAN: conditional GAN.

Model		DSC	Surface DSC	HD95/mm	MSD/mm
cycleGAN	CT-to-MV	0.89 ± 0.03	0.93 ± 0.04	1.99 ± 1.08	0.48 ± 0.10
	kV-to-MV	0.91 ± 0.02	0.94 ± 0.06	1.75 ± 0.70	0.45 ± 0.17
cGAN	CT-to-MV	0.91 ± 0.02	0.95 ± 0.03	1.38 ± 0.31	0.42 ± 0.07
	kV-to-MV	0.92 ± 0.01	0.97 ± 0.01	1.18 ± 0.20	0.36 ± 0.06

## Data Availability

The data presented in this study are available publicly at https://www.kaggle.com/datasets/yzhao15/thorax-phantoms-ctkvcbctmvcbct (accessed on 17 September 2023).
